# Associations between Zinc Deficiency, Taste Changes, and Salivary Flow Rates following Gastric Bypass and Sleeve Gastrectomy Surgeries

**DOI:** 10.1155/2024/1197571

**Published:** 2024-03-20

**Authors:** Boshra Mozaffar, Hayat Mozaffar, Mohammed Alkharaiji, Aly Elbahrawy, Iskandar Idris

**Affiliations:** ^1^MRC-Versus Arthritis Centre for Musculoskeletal Ageing Research, National Institute for Health Research Nottingham Biomedical Research Centre, Clinical, Metabolic and Molecular Physiology, University of Nottingham, Royal Derby Hospital, Derby, UK; ^2^Clinical Nutrition Department, Applied Medical Sciences, Jazan University, Jazan, Saudi Arabia; ^3^Clinical Nutrition Department, Hayat National Hospital, Jazan, Saudi Arabia; ^4^Department of Public Health, College of Health Sciences, Saudi Electronic University, Riyadh, Saudi Arabia; ^5^Department of Surgery, King Abdullah Medical City, Makkah, Saudi Arabia

## Abstract

**Background:**

The prevalence of taste change (hypogeusia) and its association with zinc deficiency is unclear due to differences in methods of assessment. We investigate the prevalence of hypogeusia using mixed methods and link it with changes in zinc levels following mini gastric bypass (MGB) and sleeve gastrectomy (SG).

**Methods:**

This was a prospective observational study of MGB (*N* = 18) and SG (*N* = 25). Hypogeusia was evaluated by using a validated questionnaire and by taste strips procedure along with serum zinc levels and salivary flow rate measurements.

**Results:**

The mean age was 40.0 ± 9.7 years; 60.5% were female. By using a questionnaire, MGB patients experienced greater hypogeusia than SG at 3 months (72.0% vs 36.0%; (*p*=0.03)), but not at 6 months (56.0% vs 45.0%; (*p*=0.74)), respectively. Using taste strips, at 6 months, more MGB patients experienced hypogeusia compared with SG (44.0% vs 11.0%; *p*=0.03). Zinc level was reduced following MGB at 6 months (85.6 ± 16.9 *μ*gm/dl vs 67.5 ± 9.2 *μ*gm/dl; (*P*=0.004)) but was increased at 6 months following SG (76.9 ± 11.4 vs 84.9 ± 21.7 *μ*gm/dl). Reduction in the rate of salivary flow was observed in 66.0% and 72.0% of MGB and SG patients, respectively, at 3 months and in 53.0% and 70.0% at 6 months.

**Conclusion:**

Taste change is more prevalent following MGB compared with SG, especially at 6 months postoperation which parallel with changes in zinc levels. More than half of all patients who had undergone bariatric surgery (BS) had low to very low salivary flow rates during the follow-up. This study suggests an association between low zinc levels and reduced salivary flow with hypogeusia following BS.

## 1. Background

Obesity has become a major health issue globally. The most recent obesity statistics in England indicate that obesity among the adult population has increased from 22.7% in 2016 to 25.3% in 2021 [1], while, according to a world health survey, in the Kingdom of Saudi Arabia, 20.2% of the adult population is now obese and 38.2% are overweight. The statistics also suggest that the number of obese women in Saudi Arabia is higher than that of men: 21.4% of Saudi Arabian women are obese and 32.7% are overweight, while 19.2% of men are obese and 42.7% are overweight [2].

Bariatric surgery (BS) has emerged as a cost-effective method to assist individuals with obesity in terms of losing weight and maintaining such loss. Globally, an estimated 468,609 BS procedures were conducted in 2013 [3], and as the prevalence of BS has grown in recent decades, the most common surgical procedures for weight reduction performed globally are Roux-en-Y mini gastric bypass (MGB) and the sleeve gastrectomy (SG) [4, 5]. In Saudi Arabia, BS is seen as one of the most prominent evidence-based options for people with morbid obesity with 15,000 bariatric procedures reported to have been carried out each year [6].

Studies, however, have reported abnormalities in taste (hypogeusia) as a typical adverse effect of BS. According to one report, 73% of patients who had MGB experienced hypogeusia [7], while another study reported hypogeusia in 64% and 59% of patients post-MGB and SG, respectively. These studies also showed that hypogeusia is associated with a higher percentage of weight loss following MGB, but not with SG [8]. The prevalence data for hypogeusia, however, are affected by differences in the methodologies used with respect to indirect vs. direct measurements. For example, in most studies utilising surveys and questionnaires, individuals self-reported changes in taste and a decline in cravings for energy-dense foods such as sweets and high-fat foods. Conversely, research studies employing proven sensory approaches, such as oral sampling or direct food intake measures, indicate little to no change in the ability to perceive taste or in evidenced preference for consuming energy-dense meals [9]. A subsequent review in 2022 also found that studies using surveys reported a considerable percentage of patients reporting experiencing taste changes following BS, while studies that assessed taste alteration using experimental approaches such as recognition thresholds, fMRI, and the sweetness acceptability test did not identify any significant effect of changes in taste after BS [10].

The precise underlying mechanism for taste alteration following BS remains unknown. Taste change can be an indicator of hypofunction of the salivary glands, which results in decreased salivary flow rates (SFRs). Saliva is crucial to the taste response, as it speeds up the transduction of tastants by solubilising and accelerating their passage to the taste pore where they attach to the receptor cells [11]. It also has lubricating and antimicrobial effects, as well as contains the growth hormones that help taste receptors regenerate [12], all of which can be affected by BS [13]. There is also evidence that zinc plays as an important element in taste bud development in healthy individuals and in saliva secretion [14, 15]. Since patients who underwent malabsorptive procedures are at a higher risk for developing zinc deficiency, the British Obesity and Metabolic Surgery Society (BOMSS, 2020) recommends targeting a ratio of 8–15 mg zinc to 2 mg copper in supplementation. The acceptable upper consumption limit for zinc, however, is 40 mg per day for those aged 19 years of age and older [6], aiming for serum or plasma zinc levels to be between 80 and 120 mcg/dL (12 to 18 mcmol/L).

Previous studies examining the prevalence of hypogeusia have used different methodologies and did not undertake concurrent assessments of zinc levels for different BS procedures. This study, therefore, aimed to assess the prevalence of GB and SG by using a mixed methods approach, applying both questionnaires and taste strips to determine the link, if any, between zinc and postoperative taste and salivary flow rate changes.

## 2. Methods

This is a single-centred prospective observational study. Consecutive subjects recruited at the obesity clinic at the hospital were scheduled for MGB and SG and were selected for the study. The main criteria for the selection of study participants were patients who have been listed for MGB and SG via registry records and through a review of medical records. Each patient participated in the study for a maximum follow-up of 6 months. This duration was selected based on likely changes in zinc levels during this time and the duration of follow-up to enable us to complete the study analysis. Recruited subjects were assessed for the following criteria: age >18 years, patients with obesity (i.e., BMI ≥35 kg/m^2^), reported failure of multiple dietary interventions designed for weight loss, and being capable of agreeing to informed consent. Exclusion criteria included subjects who were scheduled for other types of BS such as gastric banding, duodenal switch, biliopancreatic diversion, gastric balloon, and gastroplasty. We excluded patients with zinc deficiency before their surgery and/or patients who received medical prescriptions that may alter their taste function. All participants received written informed consent to be signed and dated before starting the study. The researcher explained the study details and objectives to participants and gave away detailed information sheets. Ethical approval was granted by the Institutional Review Board of King Abdullah Medical City, Ministry of Health, Kingdom of Saudi Arabia (IRB no. 20-719) in February 2021, and we started the recruitment in March 2021 and continued until August 2022. As this was an observational evaluation study, we did not perform formal power calculations.

The start of the study was when the patient attended the first study day (the same day of the surgery when the first blood sample was collected before surgery for zinc level at baseline). The second visit was at 3 months after surgery and collected blood and saliva samples. During this visit, a taste change questionnaire and taste strips' test were performed. On the third visit, we repeated the same procedure as the second visit. Patients' BMI was assessed for all study visits.

### 2.1. Study Procedure

#### 2.1.1. Serum Zinc Level

We measured the blood zinc level at baseline, at 3 months, and at 6 months following surgery. The normal level was found to be in the range of 70–120 ug/DL.

#### 2.1.2. Taste Change Assessment

- Taste change was assessed at 3 and 6 months following surgery by using the taste change questionnaire [16].
- Taste strips' test: taste strips are a validated examination procedure to examine the taste ability, and they are applied by putting them in the patient's tongue and closing the mouth. If there is interest in the gustatory sensitivity of certain tongue areas, the mouth stays open, and the strip will only be in contact with this area until the patient can provide an answer.

#### 2.1.3. Salivary Flow Rate Assessment

Stimulated saliva flow was collected by asking the participants to chew continuously on a clean square of Parafilm® for 5 minutes after an overnight fast or 2 hours after a meal. Every time the individual felt that they needed to swallow they were asked to expectorate their saliva into a sterile polypropylene graduated collection tube. Once collected, and any saliva foam had settled, the volume (in mL) was recorded, and the stimulated salivary flow rate (SFR) was determined (in mL/min). The saliva flow was classified as per the quantity of saliva as <3.5 mL (very low), 3.5–5.0 mL (low), and >5.0 mL (normal) [17]. Saliva was collected at baseline and at 3 and 6 months following the surgery.

### 2.2. Statistical Analysis

Basic descriptive statistics summarized patients' demographics, such as body weight (kg), height (cm), BMI (kg/m^2^), and zinc levels (mcg/dl) at baseline. Pearson's chi-squared statistical analysis was used to test differences in proportions between bariatric groups on their taste rankings (including their taste assessments of sweet, sour, salt, and bitter) at 3 and 6 months of follow-up. Student's *t*-test statistics were performed to obtain possible meaningful differences in means between bariatric groups regarding their zinc levels as well as their body weight reductions, compared to their baseline status, throughout the follow-up period. The paired *t*-test uses the existing data as it drops participants with no record at 6 months point to activate the “before and after” analysis.

## 3. Results

### 3.1. Patient Demographics

56 participants were included at baseline; however, 13 individuals withdrew. The reasons for withdrawal were as follows: large distance from the study site and therefore not able to attend follow-up visits (*N* = 5) and no reason provided (*N* = 8); 43 adult patients who had underwent BS at the King Abdullah Medical City facility were included in this study. Patients who underwent MGB (*N* = 18) were compared with patients who underwent sleeve SG (*N* = 25). The basic data for both the groups were as follows: female 60.47% and male 39.53%; mean age: 40.03 ± 9.7 years; total follow-up period was 6 months; and mean body weight, BMI, and zinc levels at baseline were 117.3 ± 27.4 kg, 42.7 ± 8.29 kg/m^2^, and 81.4 ± 15.0 mcg/dl, respectively. More specific patient characteristics for each group are presented in Table 1.

### 3.2. Zinc Levels and Prevalence of Hypogeusia

**Figure 1:**
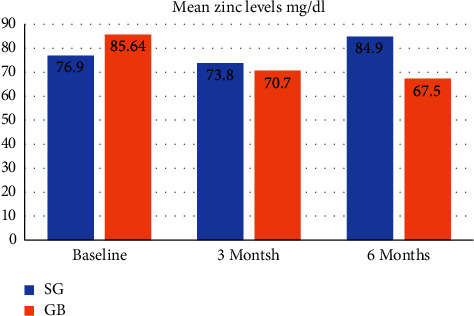
Mean zinc levels change at baseline and follow-up. A notable decline in zinc levels was seen following SG over the 3-month follow-up period, in comparison to the baseline measurements (71.4 ± 15.2 vs. 76.90 ± 11.41). Likewise, a notable reduction in zinc concentrations was seen following GB surgery throughout the 3-month follow-up period, as compared to the initial measurements (68.9 ± 12.7 vs. 85.64 ± 17.0). At the six-month mark, there was an observed rise in zinc levels among patients in the SG group (mean: 84.9 ± 21.6), whereas patients in the GB group had a further reduction in zinc levels (mean: 67.5 ± 9.16).

There was a significant decrease in zinc levels after SG and MGB at the 3-month follow-up when compared to baseline (71.4 ± 15.2 v 76.90 ± 11.41 and 68.9 ± 12.7 v 85.64 ± 17.0), respectively. At the 6-month point, zinc levels increased among SG patients (84.9 ± 21.6) but not for MGB patients (67.5 ± 9.16). Thus, overall, zinc level increased significantly (by 13.4 mg/dl) for patients who underwent SG between 3 and 6 months of follow-up (*t* (19) = −2.40; *p*=0.027), whereas zinc level remained at similar levels during the same period of follow-up post-MGB (1.44 mg/dl difference; *p*=0.59). These changes in zinc levels were parallel with the increase in the rates of hypogeusia in each case, indicating an inverse relationship between zinc levels and hypogeusia. At 6 months following SG, the mean zinc level increased significantly, reaching 84.9 ± 21.6 mg/dl; compared to 3 months, this seems to be associated with a decrease in the hypogeusia percentage (11.1% at 6 months vs 36.0% at 3 months). Zinc levels among MGB patients, however, were decreased at the 6 months of follow-up as compared to that at 3 months, and this was to parallel the increased numbers of patients experiencing hypogeusia (44.0%) (Figure 1).

The results from the taste strips' test showed that MGB patients experienced high rates of overall hypogeusia at 3 months (44.4%) and 6 months postsurgery (44.0%), which corresponded with reduced zinc levels. Sweet and salt hypogeusia occurred most frequently, and, as with SG patients, the sense of taste that did not change between the four basic taste qualities was that corresponding to bitterness 0%. However, SG patients experienced a high rate of overall hypogeusia at 3 months (36.0%) and decreased at 6 months (11.1%), which corresponded with the reduced and subsequent increase in zinc levels at 3 and 6 months, respectively. Most participants demonstrated sweet hypogeusia, with varying rates of sour, salt, and bitter hypogeusia. Hypogeusia among SG patients was found to be more frequent at 3 months and decreased at 6 months postsurgery. The sense of taste that was less affected by hypogeusia between the four basic taste qualities was that corresponding to bitterness.

**Figure 2:**
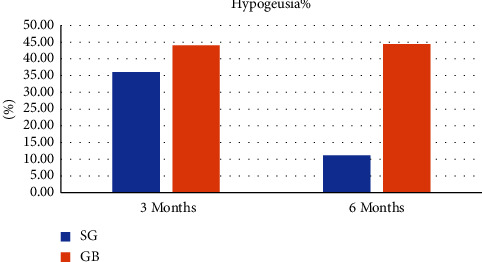
The prevalence of overall hypogeusia among SG and GB patients. At 3 and 6 months, the prevalence of hypogeusia among patients who underwent GB was higher than SG, and the percentage of hypogeusia among SG patients decreased significantly at 6 months compared to 3 months.

In total, 25 patients completed the 3-month follow-up and 20 patients completed the 6-month follow-up survey post-SG surgery, while 18 patients completed the 3-month follow-up and 17 patients completed the 6-month survey post-MGB surgery. The results of assessing both sets of data indicated that patients across both surgery procedures experienced a considerable rate of hypogeusia during the study period. However, the MGB patients were more significantly affected than the SG patients. 36% of SG patients experienced hypogeusia at 3 months, with this percentage increasing further by 6 months to 45%. In contrast, almost three-quarters (72%) of MGB patients experienced hypogeusia of taste change percentage at 3 months and 52% at 6 months. A much smaller number of patients among both the groups reported a complete loss of taste, and again the percentage among MGB patients was higher than that among SG patients. The figures for this were 8% and 10% at 3 and 6 months, respectively, for SG patients versus 33% and 31% at 3 and 6 months, respectively, after MGB. With respect to taste categories, the most notable change in taste was seen for sweet tastes, which was significant as compared to changes for the other categories (sour and salt). Most patients across both BS procedures experienced increased taste sensitivity for sweet foods, with nonsignificant higher rates of this reported at 6 months (50%) than at 3 months (75%) following SG surgery. However, while among MGB patients, the rate was higher at 3 months (58%), this decreased at 6 months (42%) (Figure 2).

Body weight and their association with zinc levels among MGB and SG patients during follow-up are provided in Table 2. Participants experienced only minor changes in responses to salt and sour tastes, with most patients reporting change experiencing an increase in salt and sour sensitivity rather than any decrease in sensitivity, as shown in Table 2.

#### 3.2.1. Correlation between Taste and Zinc Deficiency

No formal correlation between zinc deficiency and taste change was established during formal correlation analysis.

#### 3.2.2. Salivary Flow Rate

**Figure 3:**
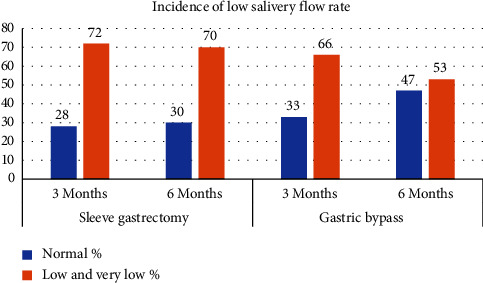
Incidence of low salivary flow rate among SG and GB patients. The salivary flow rate following both types of operations is seen to be lower than the standard range. However, there is a little rise in salivary flow rates at 6 months, with values of 4.79 ± 3.52 for SG and 4.91 ± 2.67 for GB, compared to the values reported at 3 months, which were 4.44 ± 2.84 for SG and 4.63 ± 3.03 for GB. Despite this increase, the salivary flow rates at both time points remained below the normal range.

Results in Table 3 and Figure 3 show that the mean salivary flow rate after both types of surgeries is below the normal range; while the salivary flow rates slightly increased at 6 months (4.79 ± 3.52 for SG and 4.91 ± 2.67 for MGB) as compared to those reported at 3 months (4.44 ± 2.84 for SG and 4.63 ± 3.03 for MGB). These values remained below the normal range. Zinc values were decreased at 3 months post-SG surgery, to below the normal range, though these increased to normal levels at the 6-month follow-up. MGB patients, however, experienced continuous reductions in zinc levels, which remained below normal values up to 6 months postsurgery. This tends to support an association between decreased zinc levels and decreased salivary flow rates among bariatric patients.

## 4. Discussion

Unlike reports from other studies where there was a discordant between survey and strip outcome results, the results from the survey agreed with the taste strip outcomes in this research, with a considerable percentage of patients reporting hypogeusia in the survey confirmed using the taste strip test. A comparison between the two types of surgery at 3 and 6 months showed that hypogeusia was higher among MGB patients than SG patients. These results indicate that hypogeusia is a frequent side effect after both types of BS, but more frequent among MGB patients. These results are thus in agreement with our recent review which included a variety of patient populations studied in different clinical settings that found that taste change was reported more frequently among gastric bypass patients than among those undergoing other types of BS [10]. This study also used a taste strip test to compare the outcomes after both methods of surgery: these tests confirmed that hypogeusia was more frequent after MGB than after SG. The results from previous studies that used surveys to examine taste changes following BS were thus in agreement with this: Graham, for example, found that 75% of gastric bypass patients reported taste changes [7], while Tichansky found that 82% of gastric bypass patients reported a decreased intensity of taste [18]. Our study, however, is the first to report the prevalence of hypogeusia following a MGB operation.

No studies using patient self-reports contradict this idea of taste change following BS, though controversy has arisen based on variance in detecting taste changes using quantitative or experimental methods such as taste detection and recognition thresholds. A recent review suggested that the discordant between these studies was due to methodologies employed to measure taste alteration. While survey research that sees patients regularly reported changes in taste after BS, commonly 6 months after surgery, and research studies using experimental techniques to investigate taste alterations, such as recognition thresholds, fMRI, and the sweetness acceptability test, have not generally discovered any appreciable rate of change in taste after BS (10). In contrast to previous experimental studies that did not detect any change in taste, our work showed that a considerable number of participants demonstrated hypogeusia based on the taste strips' examination test.

In parallel with hypogeusia, we observed a significant corresponding reduction in mean zinc levels among MGB patients from baseline and after 6 months, despite multivitamin supplementation being taken by patients every day. In this study, the surgeons prescribed centrum supplementation for at least 6 months following sleeve gastrectomy operations, and for life after MGB surgeries. However, the single tablet serving for this supplement contains only 5 mg of zinc, an amount significantly less than the recommended daily intake according to the British Obesity and Metabolic Surgery Society (BOMSS, 2020), which suggests that zinc should be consumed at a ratio of 8–15 mg per mg of copper [19]. The maximum daily zinc consumption that is unlikely to have a negative impact on health is 40 mg for those over 19 years of age [20], and the role of zinc in the support of taste and the development of taste buds has been well-established in the previous literature [21, 22]. These facts, taken together, suggest that the current zinc supplementation prescribed in clinical practice is insufficient to avoid zinc deficiency and thus to help prevent taste change following MGB. Currently, no study has previously investigated the dosage of zinc supplementation and its effect on taste change in MGB patients.

This study supports a possible link between zinc deficiencies and hypogeusia following BS. The most interesting finding from this study may be, however, that the percentage of hypogeusia, while high among SG patients at 3 months, significantly decreased in SG patients at 6 months postsurgery, which is parallel to the increase in zinc levels seen in such patients during the same period. Similarly, the continuous decrease in the sense of taste at 3 and 6 months after MGB appears to be associated with persistence in a deficiency in zinc levels, further supporting zinc level as an important determinant of hypogeusia. The higher taste change percentage among MGB patients may thus be explained by the reduction in the intestinal absorption of consumed food bypassing a portion of the small intestine and up to 90% of the stomach, leading to the consumption of less food and the deactivation of the portion of the intestine where sugar and fat are mainly ingested [23]. While zinc is absorbed throughout the entire small intestine, this occurs mainly in the duodenum and jejunum [24].

To the best of the author's knowledge, this is the first clinical study examining the possible relationship between zinc deficiency and taste changes following BS. The majority of patients receiving both types of surgeries experienced an increase in sweet taste intensity, with the incidence of this being higher at 6 months (35%) than at 3 months (24%) after SG surgery. However, among MGB patients, while the incidence of this was higher at 3 months postsurgery (38%), this was significantly decreased at 6 months (18%). The precise cause of this increase in sweet taste intensity also remains unclear, and is a topic for further research. However, the fact that taste buds can only detect flavours within moisture may make a difference here: those with dry tongues cannot taste dry substances, while increased viscosity reduces taste sensitivity [11]. Salivary issues might thus be another cause of taste change following BS. The study findings did suggest that more than half of the study participants experienced low to very low salivary flow rates after both types of BS. This observation was discordant with a recent systematic review and meta-analysis that evaluated salivary flow changes after bariatric surgery which reported no significant alteration in salivary flow rates for up to 24 months after bariatric surgery [25]. In this case, however, the rate of affected patients was higher at 3 months postsurgery, slightly decreasing at 6 months; this further coincided with the decrease in zinc levels. Zinc plays an important role in developing taste buds and increasing salivary flow and it is thus suggested that reduced zinc levels following BS may cause a reduction in salivary flow secretions that further promote hypogeusia or taste change.

Some limitations need to be highlighted. Importantly, this study was conducted throughout the COVID-19 pandemic, and taste change in some patients may thus have been affected by COVID-19 symptoms, based on the fact that it was not possible to do a correlation test between zinc levels and taste changes. However, the patient was tested for COVID-19 using PCR before BS. Additional functional methods to assess taste change would have improved the sensitivity and specificity of our study's findings. This study was only conducted in a single centre and hence findings may not be able to be generalised to all clinical settings. Finally, the bypass operations undertaken in this study were entirely mini bypass surgery rather than the traditional Roux-en-Y gastric bypass, and the latter was anticipated to be associated with more significant zinc deficiency and potentially increased risks of hypogeusia due to the greater amount of absorptive surfaces excluded.

To conclude, zinc levels among MGB patients were lower than SG and corresponded with an increased prevalence of hypogeusia. Both methods used to assess taste change confirmed the presence of taste change following BS. More than half of the patients across both surgery types had low to very low salivary flow rates which may also contribute to the high prevalence of hypogeusia post-BS. Since, this is a single-centre study, we have to caution on the generalizability of these findings to other populations but we believe that the study has enhanced our understanding of the mechanistic link between hypogeusia and zinc levels in MGB and SG procedures. A further multicentred study will be required with longer-term, follow-up to determine the relationship between zinc, hypogeusia, and MGB patients.

## Figures and Tables

**Table 1:** Patient characteristics at baseline.

	Sleeve gastrectomy (*N* = 25)	Mini gastric bypass (*N* = 18)
Age, mean (SD)	38.12 (11.58)	41.88 (7.41)
BMI, mean (SD)	45.90 (6.63)	39.46 (8.68)
Body weight kg, mean (SD)	125.9 (23.57)	108.95 (28.60)
Zinc mg/dl, mean (SD)	76.90 (11.41)	85.6 (16.9)

**Table 2:** Mean differences for weight reduction, zinc levels, taste strips, and questionnaire outcomes at 3- and 6-month follow-ups.

	3 months		Diff.^†^	6 months		Diff.
	SG (*n* = 25)	GB (*n* = 18)		SG (*n* = 20)	GB (*n* = 17)	
BMI reduction kg/m^2^, mean (SD)	8.52 (5.9)	4.35 (3.4)	0.01	10.9 (5.7)	6.0 (3.8)	0.005
Body weight reduction kg, mean (SD)	20.8 (7.1)	13.4 (7.1)	0.001	30.6 (16.1)	17.9 (8.8)	0.006
Zinc mg/dl, mean (SD)	71.4 (15.2)	68.9(12.7)	0.51	84.9 (21.6)	67.5(9.16)	0.004

*Questionnaire elements n (%)*						
Appetite change, *n* = 43	18 (72.0%)	13 (72.2%)	0.98	17 (85%)	10 (63%)	0.15
Taste change, *n* = 43	9 (36%)	13 (72%)	0.03	9 (45%)	9 (56%)	0.74
Smell change, *n* = 42	4 (17%)	4 (22%)	0.70	3 (15%)	4 (25%)	0.68
Overall loss of taste, *n* = 43	2 (8%)	6 (33%)	0.05	2 (10%)	5 (31%)	0.20
Increased taste to sweet foods, *n* = 20	6 (75%)	7 (58%)	0.64	7 (50%)	3 (42%)	0.99
Decreased taste to sweet foods, *n* = 20	2 (25%)	6 (50%)	0.37	2 (14%)	2 (29%)	0.57
Increased taste to salty food, *n* = 19	4 (50%)	4 (36%)	0.65	1 (8%)	0 (0%)	—
Decreased taste to salty foods, *n* = 19	0 (0%)	2 (18%)	—	2 (15%)	1 (17%)	0.99
Increase taste to sour foods, *n* = 18	4 (16%)	3 (17%)	0.63	3 (23%)	2 (33%)	0.99
Decreased taste to sour foods, *n* = 18	1 (13%)	4 (40%)	0.31	3 (23%)	0 (0%)	—

*Taste strips' outcomes*						
Overall hypogeusia, *n* (%)	9 (36.0)	8 (44.4)	0.75	2 (11.1)	7 (44.0)	0.052
Sweet hypogeusia, *n* (%)	6 (24.0)	6 (33.3)	0.51	2 (10.5)	2 (12.5)	0.86
Sour hypogeusia, *n* (%)	4 (16.0)	5 (27.8)	0.45	3 (15.8)	5 (31.3)	0.42
Salt hypogeusia, *n* (%)	5 (20.0)	6 (33.3)	0.48	4 (21.1)	4 (25)	0.78
Bitter hypogeusia, *n* (%)	3 (13.6)	0 (0)	—	0 (0)	0 (0)	—

^†^The test for a difference was performed by using an independent *t*-test for parametric variables. Person's chi-square test was used for the difference in proportions, with a *P* value of 0.05, while the ^†^ Fisher's exact test for low expected frequencies was used for the taste change questionnaire outcome (i.e., 20% of the expected frequencies at <5), with a *P* value of 0.05. ^†^Fisher's exact test was also used for the outcomes of the taste strips' test to illustrate the % of overall hypogeusia and % of hypogeusia in each taste quality during the follow-up period, and among patients who underwent sleeve gastrectomy and gastric bypass. Patients are subject to sleeve gastrectomy (SG) and gastric bypass (GB).

**Table 3:** Mean salivary flow rates and zinc levels among sleeve gastrectomy and gastric bypass patients during the follow-up period.

	Gastric bypass (*N* = 18)		Sleeve gastrectomy (*N* = 25)	
	3 months *n* = 18	6 months *n* = 17	3 months *n* = 25	6 months *n* = 20
Salivary flow rate ml/min mean (SD)	4.63 (3.03)	4.91 (2.67)	4.44 (2.84)	4.79 (3.52)
Salivary flow rate (low and very low), %	6 (33%)	8 (47%)	18 (72%)	14 (70%)

## Data Availability

The data used to support the findings of the study are available from the corresponding author upon request.

## Additional Points

*Highlights*. Both mini gastric bypass (MGB) and sleeve gastrectomy (SG) were associated with increased rates of hypogeusia postsurgery but the MGB procedure led to a significantly higher rate of hypogeusia than SG at 3 months (72.0% vs 36.0%; *p*=0.03 using a questionnaire) and at 6 months (44.0% vs 11.0%; *p*=0.03 using taste strip). There were minor differences in the outcomes between questionnaire and taste strip assessments of hypogeusia but both methods concluded a higher rate of hypogeusia with MGB than with SG. A higher rate of hypogeusia was associated with reduced zinc levels among MGB patients. Both procedures lead to a reduction in salivary flow rate in more than 50% of patients, which may also contribute to the prevalence of hypogeusia with both surgical procedures. We concluded an association between low zinc levels and reduced salivary flow with hypogeusia following bariatric surgery, the latter being more prevalent following MGB compared with SG.
